# The Design and Development of a Microstrip Antenna for Internet of Things Applications

**DOI:** 10.3390/s23031062

**Published:** 2023-01-17

**Authors:** Liliana Anchidin, Alexandru Lavric, Partemie-Marian Mutescu, Adrian I. Petrariu, Valentin Popa

**Affiliations:** Computers, Electronics and Automation Department, Stefan cel Mare University of Suceava, 720229 Suceava, Romania

**Keywords:** IoT devices, wireless sensor networks, IoT applications, small patch antenna, SigFox

## Abstract

The Internet of Things (IoT) has become a part of modern life where it is used for data acquisition and long-range wireless communications. Regardless of the IoT application profile, every wireless communication transmission is enabled by highly efficient antennas. The role of the antenna is thus very important and must not be neglected. Considering the high demand of IoT applications, there is a constant need to improve antenna technologies, including new antenna designs, in order to increase the performance level of WSNs (Wireless Sensor Networks) and enhance their efficiency by enabling a long range and a low error-rate communication link. This paper proposes a new antenna design that is able to increase the performance level of IoT applications by means of an original design. The antenna was designed, simulated, tested, and evaluated in a real operating scenario. From the obtained results, it ensured a high level of performance and can be used in IoT applications specific to the 868 MHz frequency band.By inserting two notches along x axis, we find an optimal structure of the microstrip patch antenna with a reflection coefficient of −34.3 dB and a bandwidth of 20 MHz. After testing the designed novel antenna in real IoT operating conditions, we concluded that the proposed antenna can increase the performance level of IoT wireless communications.

## 1. Introduction

The Internet of Things (IoT) has become a part of modern life where it is used for data acquisition and long-range wireless communications. The IoT concept is usually implemented by means of sensors that are able to transmit information in a wireless network configuration. Applications based on wireless sensor networks (WSN) technologies range from healthcare systems, the military, smart city, and smart homes to agricultural applications [1,2,3,4,5,6,7] focused on increasing efficiency and improving resource allocation.

This IoT penetration is sustained by various wireless communication protocols that enable data transmission such as: Bluetooth (BLE) [8], LoRa (long range) modulation [9], SigFox [10] or 5G [11] technologies.

Regardless of the IoT application profile, every wireless communication transmission is enabled by highly efficient antennas. The role of the antenna is very important and must not be neglected. The antenna must be easy to manufacture, have small dimensions, be lightweight, have a low manufacturing cost, and be compatible with different integrated-circuit configurations. A microstrip patch antenna (MPA) can achieve these constraints and be easily designed for different configurations. Different types of microstrip patch antennas can be designed in various geometric forms such as rectangular, square, or circular [12]. Considering the high demand of IoT applications, there is a constant need to improve antenna technologies, including new antenna designs, in order to increase the performance level of the WSNs and increase communication efficiency.

The main challenges of the IoT sensors are related to the following issues:

- IoT sensors are usually battery-powered and the low-energy consumption is an important constraint [13];
- The communication environment is characterized by urban non-LoS (Line of Sight) propagation conditions and the antenna must enable a low error-rate communication link;
- The communication range must be as large as possible in order to ensure connectivity over large geographical areas such as the surface of a city;
- Wireless communication protocols must coexist since many of the IoT technologies operate in unlicensed communication frequency bands [14].

IoT applications require a low-consumption profile and more efficient antennas that can improve the performance level of the communication link, as well as provide a long-range information transfer. Many of the IoT communication protocols operate in the ISM (Industrial, Scientific, and Medical) and SRD (Short-Range Devices) non-licensed frequency bands, where the 868 MHz band is the one most commonly used by LoRa and SigFox IoT communication protocols. The designed IoT antenna must therefore ensure a high level of performance in the 868 MHz frequency band.

In this paper, we make the following contributions:

- the design, development and test of a highly efficient antenna that is suitable for IoT applications;
- the antenna is produced using an FR-4 substrate, is low-cost, and relatively easy to manufacture;
- the novel design can be directly integrated into IoT devices operating in the 868 MHz frequency band;
- the performance level of the antenna was evaluated using laboratory equipment and a real operating scenario, being compared with two reference IoT antennas;
- from the obtained results we concluded that the proposed antenna ensures a high level of performance and can increase the efficiency of SigFox communications.

The paper is organized as follows: first, a brief introduction related to the state-of-the-art, followed by Section 2, where the main antenna design for IoT challenges is presented in detail. The main goal was to obtain the highest level of performance for IoT communications. In Section 3, the performance level of the designed IoT antenna is presented and discussed in detail. The antenna was evaluated in real operating conditions and different performance metrics were recorded and analyzed.

The final section of the paper consists of the conclusions and the overall performance discussion of the developed antenna. From the obtained results presented in this paper, we conclude that the proposed antenna design ensures a high-level performance in the 868 MHz frequency band and can be easily integrated into many IoT applications.

## 2. IoT Technologies and Antenna Design

In recent years, the research interest in wearable antennas inserted on flexible materials [15,16,17] has increased. Biomedical sensor networks for health care applications have drawn great attention mainly due to the recent pandemic context; therefore, wireless IoT communication systems with large range communication capabilities are in high demand. When evaluating the performance level of IoT technologies, another important aspect that should not be neglected is related to the communication protocols integrated at the application level.

In this paper, we focus our attention on Sigfox communication protocol that is a Low- Power Wide-Area Network type used for IoT applications in the free-licenses SRD 868 MHz frequency band. To evaluate the quality of a system, three major criteria are considered, i.e., the communication range, the communication speed and the power consumption needed to provide the first two. Sigfox addresses these problems with some advantages: low power, long range capability and high robustness to interferences. Another aspect is related to the dimensions of the antenna that must be reduced and provide support for small-form wireless sensors integration. An electrically small antenna is defined as an antenna area equal or less than 0.1λ [18].

Due to the low dimensions of WSNs modules, microstrip antennas are usually preferred in many IoT applications. A microstrip antenna is characterized by its small size and a narrow frequency-band behavior antenna [19]. Low radiation efficiency usually resumes to a decreased gain. An improvement on bandwidth of the antenna structure can be achieved by increasing the width using a high dielectric substrate, introducing specific slots [20] or notches on the patch, or by increasing the number of propagation modes of the antenna.

As shown by Chu [19], small dimension antennas usually have a narrow frequency bandwidth because of a high Q-Factor parameter. In the literature, several works have used matching circuits to increase the frequency bandwidth [16].

**Figure 1:**
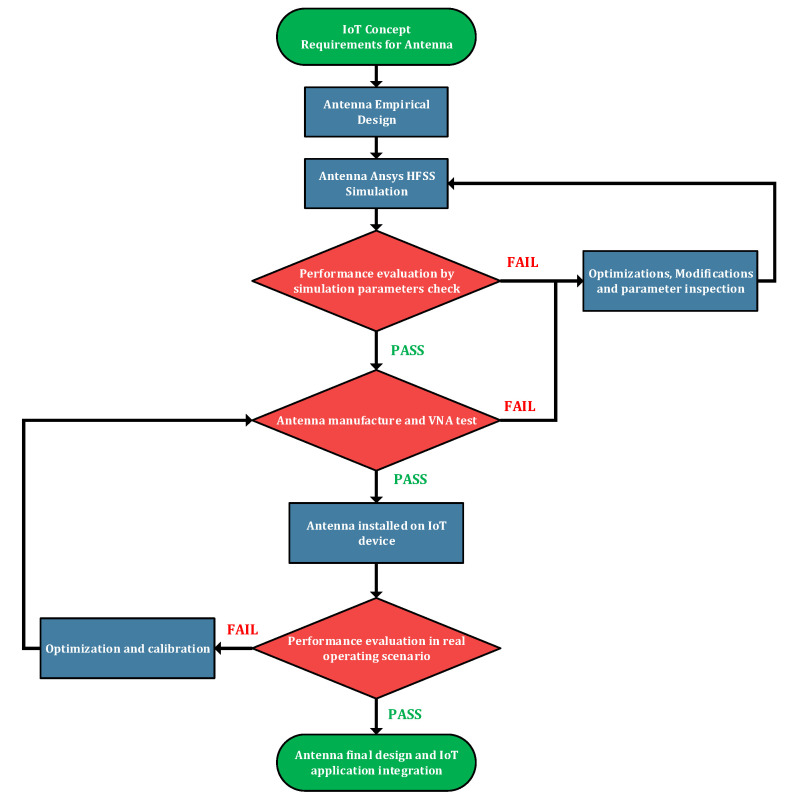
Design methodology.

Figure 1 presents the design methodology used for the developed IoT antenna. The first step was to analyze the IoT concept antenna requirements, empirically design the antenna, and evaluate its performance level through simulations by performing optimizations, modifications and parameter inspections. The next step was to manufacture the selected configuration and test it using laboratory equipment to perform calibrations. The final step included the integration of the designed antenna in a real IoT application and evaluating the level of performance.

In order to increase the radiated power and to decrease the input impedance, we increased the substrate height; consequently, the radiation resistance was approximately 300 Ω [21]. Another technique to decrease the input impedance is to increase the width dimension of the antenna structure.

There are some methods to increase the performance level such as: short-circuits, increasing electrical length [22], using magnetic or high permittivity substrates, and using superstrates or a combination of them in order to design electrically small antennas that are able to operate at high frequencies.

Another concern about the antenna is the excitation method of the patch, i.e., transmission line, probe feed and aperture coupling. When a transmission line is used, the energy is converted by Joule effect into heat and an aperture coupling increases the difficulty of the practical implementation. The feeding point must be placed at a distance at least equal to one third of the distance between the two slots; however, the location point should be adjusted where the antenna input impedance is equal to the characteristic impedance of the connecting coaxial cable in order to comply with the impedance’s match. In this paper, we focused on the design of a microstrip patch antenna for long-range IoT communications protocols that are suitable for urban non-LoS (Line of Sight) up to 2 km [23].

## 3. The Design, Development, and Implementation of the IoT Antenna

In the literature there are many microstrip antenna design techniques [24,25,26] that use the cavity model and transmission line model because of their low complexity. Considering that the ratio height (h) over width (W) is very small for our antenna design requirements, the current densities moving around the principal conductor can be achieved at the edges of the patch antenna. Furthermore, since the substrate thickness is very small compared to the dielectric wavelength (h << λ_g_), the electric field can be considered normally distributed on the patch surface. In that case, we considered that the microstrip antenna could be modeled as a resonant cavity where the top and bottom are electrical walls, and the sides are magnetic walls [26,27].

**Figure 2:**
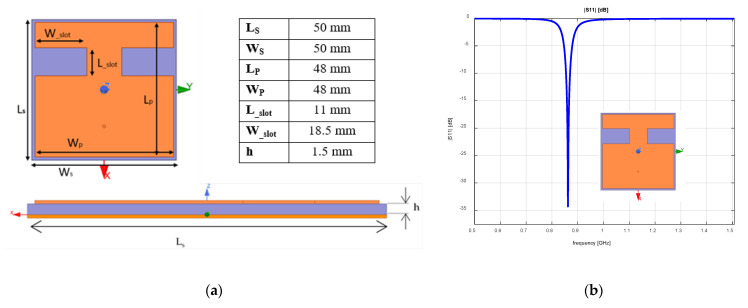
Antenna dimensions: (**a**) Front view and side view with dimensions; (**b**) Reflection coefficient.

In this section, we present the design and development of the proposed antenna considering different IoT application requirements specific to the 868 MHz frequency band. The proposed microstrip patch antenna (Figure 2a) was manufactured on an Fr-4 substrate with a relative permittivity of 5.1 and a loss tangent parameter of 0.02. The substrate height was 1.5 mm and the thickness of the antenna copper was about 0.035 mm. In order to achieve the resonant frequency for a patch antenna of 50 mm × 50 mm, two notches were inserted in the structure along the x axis with the role of increasing the electrical length of the patch. The antenna’s size was 0.13λ × 0.13λ, with the substrate and the ground plane dimension of 0.14λ × 0.14λ. Antenna size was reduced by 1 mm in respect to the ground in order to increase the active power output.

The entire structure of the antenna was simulated using the Ansys HFSS [28] environment to verify the influence of the notches on the electrical parameters of the antenna. Figure 2b represents the reflection coefficient of the proposed antenna with the value at 868 MHz of −34.3 dB. This section presents in detail the design choices that led to the antenna with the above-presented dimensions. The design steps of the IoT antenna included the determination of the optimal influence of the notches’ width and length on the antenna parameters, the evaluation of the notches’ position, the feeding point location in respect with the reference center and the overall dimensions of the antenna.

We verified the influence of the notches’ width; the evaluated values were 16, 17, 18.5, 20 and 21 mm. At 16 and 17 mm, the resonant frequencies achieved 0.98 GHz and 0.94 GHz, respectively, and for 20 and 21 mm, the resonant frequency decreased to 0.79 GHz and 0.74 GHz, respectively.

**Figure 3:**
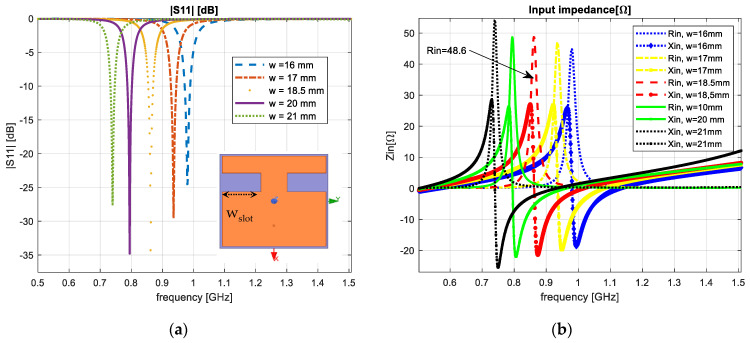
Varying the notches’ width: (**a**) reflection coefficient; (**b**) input impedance.

Figure 3 shows the variation of the reflection coefficient (a) and input impedance (b), respectively, when the notches’ width dimension varied.

**Figure 4:**
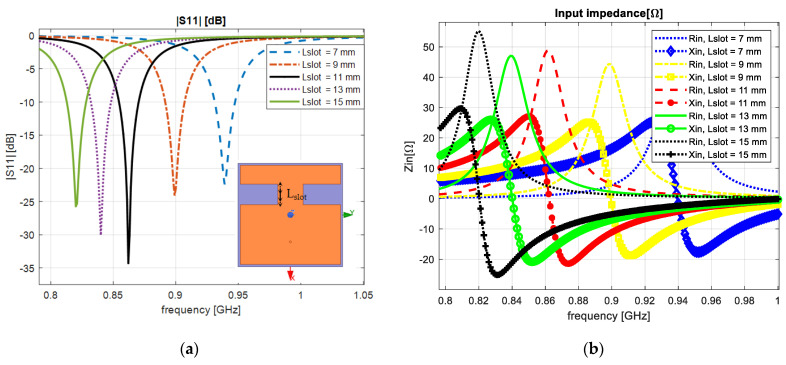
Varying the notches’ length: (**a**) reflection coefficient; (**b**) input impedance.

The reflection coefficient for the notches’ length size of 7, 9, 11, 13 and 15 mm is presented in Figure 4a. For 7 and 9 mm, we obtained a resonant frequency of 0.94 GHz and 0.9 GHz, and for 13 and 15 mm we achieved 0.84 GHz and 0.82 GHz. In this particular case, the real part of the input impedance achieved 42.69 Ω, 44.13 Ω, 46.89 Ω, 48.6 Ω and 54.96 Ω as shown in Figure 4b.

From the obtained results, we observed that when increasing and decreasing the size of the notches, we varied the electrical length of the patch antenna. We achieved an optimal antenna dimension for the size of 18.5 mm × 11 mm with a reflection coefficient of −34.3 dB.

**Figure 5:**
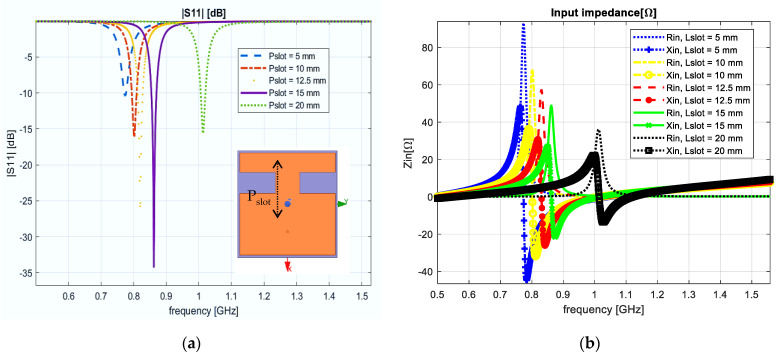
Patch antenna with two notches. The slot position is moved along the x axis. (**a**) Reflection coefficient; (**b**) input impedance.

In the second evaluation scenario, we simulated the influence of the notches’ position on the antenna’s principal parameters. The position was moved in respect with the axial direction of propagation. From the obtained results, the notches’ size dimensions were set to 18.5 mm × 11 mm. When the two notches move further from one another, the resonant frequency increases. The variation was 5, 10, 12.5, 15 and 20 mm, respectively, from the center of the reference. Figure 5a shows the reflection coefficient variation from 0.77 GHz to 1.01 GHz, and Figure 5b shows the input impedance.

**Figure 6:**
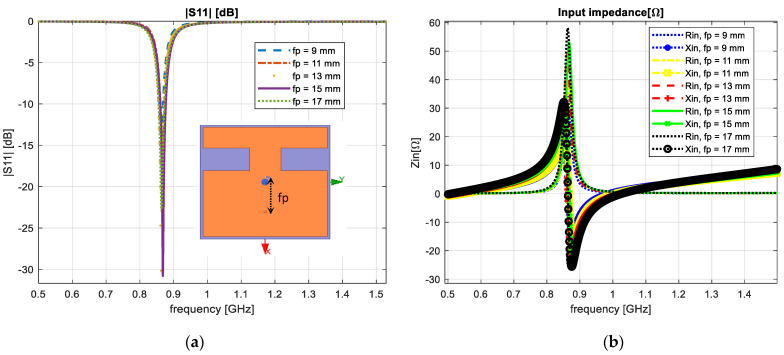
Feed-point position variation: (**a**) reflection coefficient; (**b**) input impedance.

For a small size antenna, the input impedance is around 300 Ω. In order to decrease this value, we had to find the optimal voltage-to-current ratio. We varied the feed-point position to 9, 11, 13, 15 and 17 mm, respectively, from the center of the antenna, with the aim to set the match between the input impedance and the cable connection input. The obtained results are presented in Figure 6.

From the obtained results, we achieved an optimum value of the input impedance parameter when the feed-point was set at 13 mm from the center of the developed IoT antenna.

**Figure 7:**
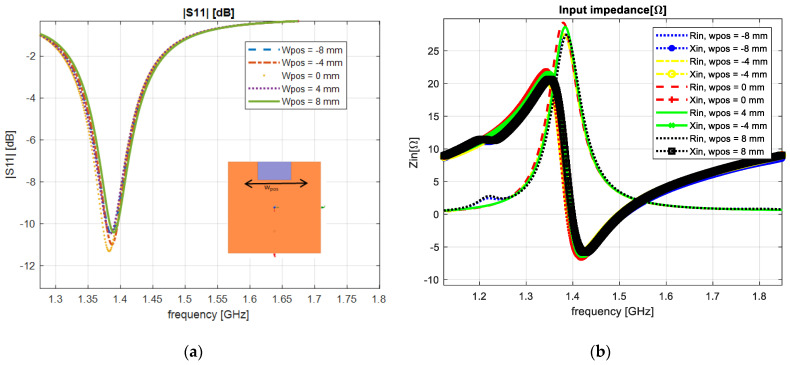
Varying the notches’ position: (**a**) reflection coefficient; (**b**) input impedance.

The next step was to evaluate the antenna with a notch inserted in the front part of the antenna. The notch position was modified along the Y axis with different positions (−8, −4, 0, 4, 8 mm with respect to the center of the references), in order to evaluate the highest level of performance. In Figure 7a, the reflection coefficient parameter achieved a value of −11.18 dB, and the upper input impedance parameter a value of 27 Ω, respectively, as seen in Figure 7b.

**Figure 8:**
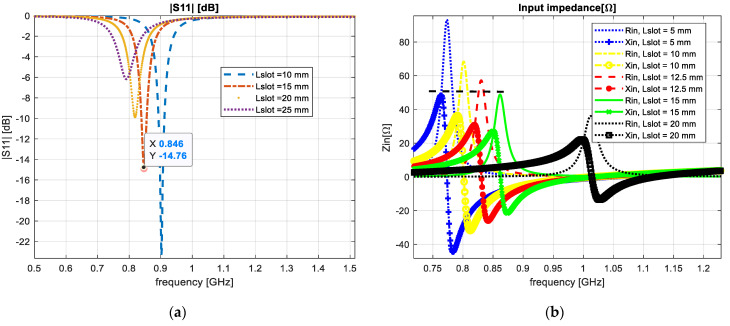
A centered slot integrated in the antenna design: (**a**) reflection coefficient; (**b**) input impedance.

The next design step was to insert an interior slot of rectangular form that would be able to increase the antenna electrical length, as presented in Figure 8a. We simulated the structure with different dimensions and the slot was moved along the x axis of the patch with a distance of 10, 15, 20 and 25 mm.

Figure 8b shows a reflection coefficient parameter of $\left|S_{11}\right|≅-24\;\mathrm{dB}$ and an input impedance of 55 Ω at a central frequency of 0.9 GHz.

**Figure 9:**
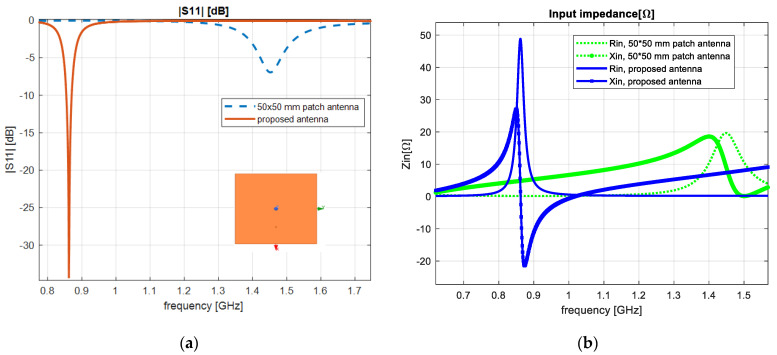
Rectangular patch antenna: (**a**) reflection coefficient; (**b**) input impedance.

A microstrip patch antenna with 50 mm × 50 mm × 0.035 mm size was evaluated, as presented in Figure 9. The structure was realized on an FR4 substrate with 50 mm × 50 mm × 1.5 mm. The patch and the ground were connected to a probe-feed by an inner and an outer conductor, respectively. From the obtained results, we observed that the ground-plane connection ensured a high level of performance and was thus integrated in the antenna design.

**Figure 10:**
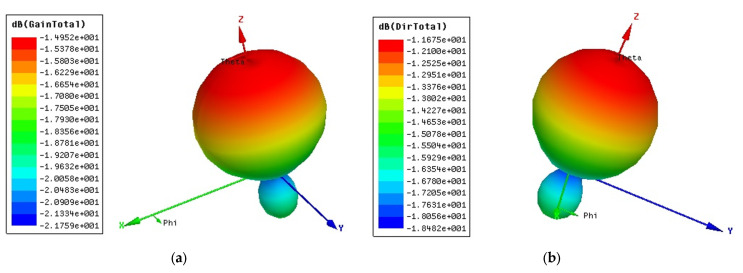
The radiation pattern of the developed IoT antenna: (**a**) gain and (**b**) directivity.

Figure 10a shows the radiation pattern of the designed antenna; the gain value was around −1.49 dB, while the directivity is presented in Figure 10b and achieved a maximum value of −1.16 dB. The radiation efficiency parameter of the designed IoT antenna achieved a value of 51%.

**Figure 11:**
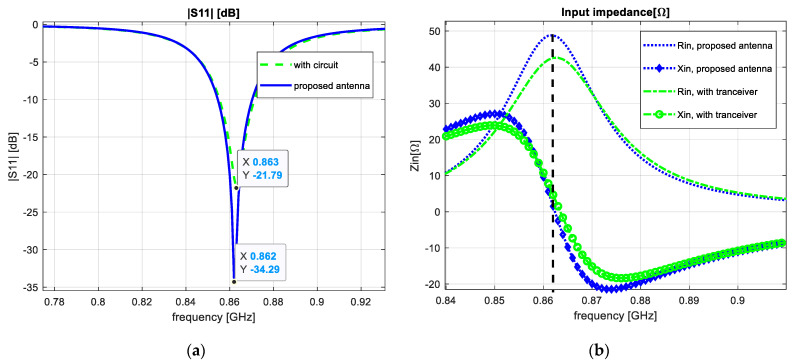
Transceiver influence: (**a**) reflection coefficient; (**b**) input impedance.

One final step was to analyze the influence of the circuit layer over the antenna parameters. The transceiver placed on the underground plane did not affect the resonant frequency because the size of the ground plane was a smaller dimension compared to the antenna size. We can observe in Figure 11 that, by placing the circuit under the plane, the reflection coefficient decreased to −21.79 dB. The real part of the input impedance decreased to 42.41 Ω and we achieved a VSWR parameter of approximately 1.18.

## 4. Performance Evaluation of the Proposed IoT Antenna

**Figure 12:**
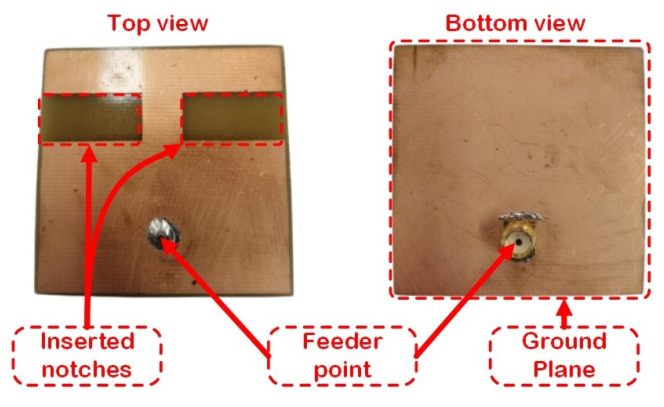
The developed IoT antenna.

In this section, we present the performance evaluation of the designed IoT patch antenna. The antenna was tested in a real operating environment and its performance was compared with two reference antennas: one omnidirectional and one microstrip, both designed for the 868 MHz frequency band. Figure 12 presents the developed IoT antenna that was further tested in real operating conditions.

As a reference for evaluation purposes, we used an omnidirectional antenna rated for the 868 MHz frequency band included in the ON Semiconductors Sigfox development KIT with a gain of 1 dBi, and a patch antenna respecting the inverted F design [29] with a gain of 0 dB, also rated for the 868 MHz frequency band.

**Figure 13:**
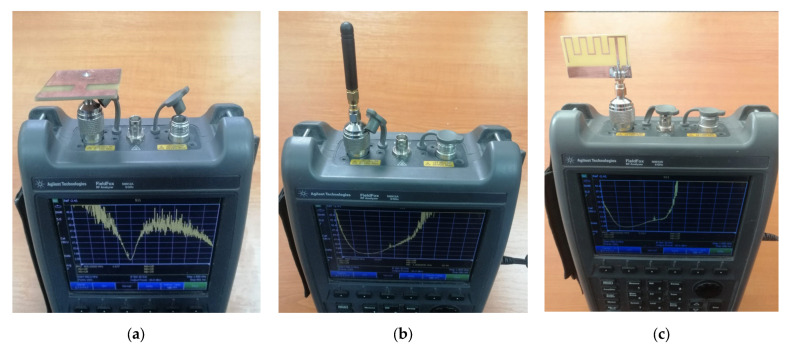
The proposed test setup: (**a**) proposed novel IoT antenna; (**b**) reference omnidirectional antenna; (**c**) reference microstrip antenna.

The first step was to perform a vector network analyzer (VNA) analysis of the antennas, in which we measured the S11 parameter in terms of reflection coefficient and VSWR (Voltage Standing Wave Ratio), for frequencies ranging from 500 MHz to 1.5 GHz. Figure 13 presents in detail the test setup used.

From the obtained results, in the 868 MHz frequency band, the omnidirectional reference antenna achieved an average reflection coefficient of −2.08 dB and a VSWR value of 8.78; the microstrip reference antenna achieved an average reflection coefficient of −1.81 dB and a VSWR value of 9.6; while the proposed novel IoT antenna achieved an average reflection coefficient of −12.8 dB and a VSWR value of approximately 1.6.

**Figure 14:**
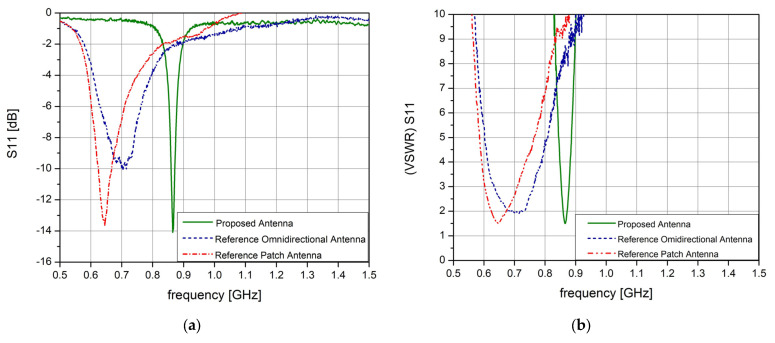
Test results: (**a**) reflection coefficient; (**b**) VSWR.

In Figure 14, we observe the reflection coefficient of the reference antennas and the novel designed IoT antenna, as well as the VSWR parameter. The omnidirectional and microstrip reference antennas each present one minimum value around the 704 MHz and 645 MHz frequency mark with the values of −10.13 dB and −13.74 dB, respectively, while our proposed antenna presents one minimum value around the 865 MHz frequency mark with a value of −14.08 dB; all the antennas were rated for the 868 MHz frequency band.

In Figure 14b, we see that the reference antennas have a wideband frequency behavior, with a VSWR value below 10 in the frequency range of 570 MHz to 900 MHz with an absolute minimum VSWR value of 1.9 around the 715 MHz frequency mark for the omnidirectional antenna, and a minimum VSWR value of 1.51 around the 645 MHz frequency mark for the reference patch antenna, while the proposed antenna has a narrowband behavior, with an absolute minimum VSWR value of 1.49 around the 865 MHz frequency mark. These characteristics prove without a doubt that our designed IoT antenna performed better for IoT specific applications.

**Figure 15:**
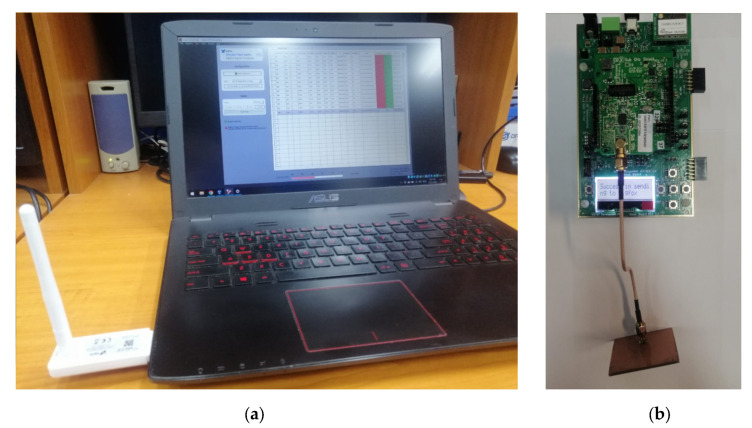
Real operating scenario test setup: (**a**) Sigfox SDR certification Kit with the SigFox Radio Signal Analyzer App; (**b**) ON Semiconductors Sigfox Development Kit with the proposed antenna attached.

The next step was to further evaluate the IoT-designed antenna in a real operating scenario for Sigfox communications, as shown in Figure 15. Sigfox [30] is an ultra-narrow band LPWAN (Low Power Wide Area Network) communication protocol, operating in the 868 MHz frequency band on multiple 100 Hz communication channels.

The main advantage of Sigfox communication protocol is its redundancy mechanism in which a single message is sent three times, at three different timestamps, on three different communication channels, ensuring diversity both in time and frequency.

The RSSI (Received Signal Strength Indicator) is a parameter that indicates the quality of a radio link between the transmitter and the receiver and is a measure of the performance level. Therefore, the higher the RSSI values, the stronger the power of the radio signal. Although the SigFox receiver selects the packet with the highest RSSI at the receiving end, the arrival of all three data packets points to a high-quality link that corresponds to the best-case scenario.

**Figure 16:**
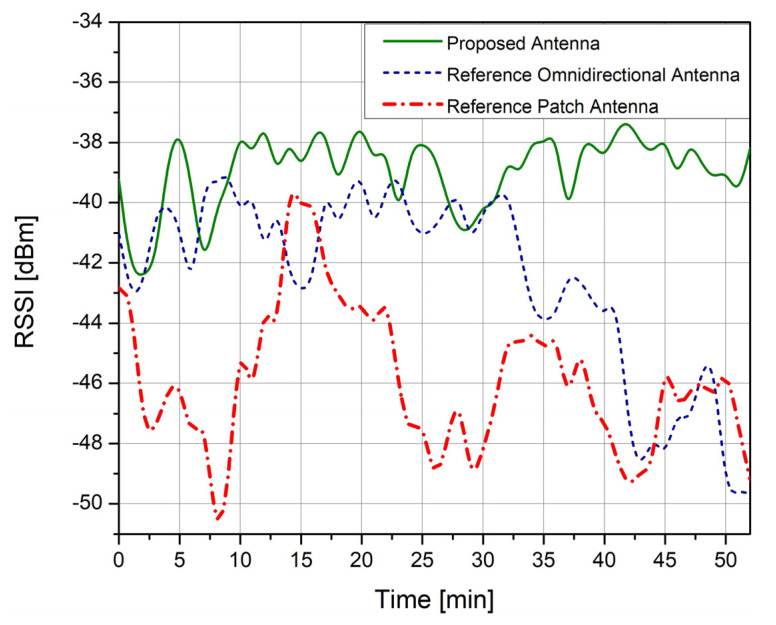
RSSI parameter variation obtained in the real operating scenario.

For the real operating scenario test setup, we used the Sigfox SDR certification kit [31] and the ON Semiconductors Sigfox transceiver, as presented in Figure 16. The tests included three different scenarios, two where the SigFox ON transceiver used the reference antennas and one scenario that included the use of the proposed IoT antenna mounted on the SigFox transceiver.

The evaluation was performed in a non-line of sight (non-LoS) propagation conditions indoor scenario that included a distance of approximately 30 m between the receiver and the transmitter. We used the Sigfox SDR kit to monitor the received data packets while at the same time recording the RSSI parameter of each individual packet. From the obtained results, the reference omnidirectional antenna achieved an average RSSI of −44.56 dBm, the reference patch antenna achieved an average RSSI of −46.56 dBm, and the proposed novel IoT antenna achieved an average RSSI of −38.56 dBm. Figure 15 presents the RSSI parameter variation for the reference antennas and the novel designed IoT antenna. From the obtained results, we see that the proposed IoT antenna had the highest performance level.

## 5. Conclusions

In this paper, we present the design, development, and performance evaluation of a microstrip patch antenna with small sizes for Internet of Thing applications. The antenna was integrated in a SigFox transceiver, designed to operate at 868 MHz. For this frequency, antennas have large sizes, i.e., L = 82.15 mm and W = 105.1 mm; in this case, we introduced two notches on the axial direction of propagation with the aim to increase the electrical length of the patch. By inserting two notches along the x axis, we found the microstrip patch antenna to be an optimal structure with a reflection coefficient of −34.3 dB and a bandwidth of 20 MHz.

The antenna was manufactured on an FR-4 substrate with electrical permittivity, εr = 5.1 and loss tangent, tanσ = 0.02. The antenna was placed on the upper side of the configuration, and the ground plane on the bottom side of the configuration. The final antenna had a square form of 48 mm × 48 mm with a thickness of 0.035 mm and a substrate high of 1.5 mm with two notches inserted on both sides. The feeding point location varied in order to determine the optimum point for matching the input impedance and co-axial cable impedance. After the antenna was designed and simulated, it was physically produced and tested in a real IoT operating condition.

From the obtained results, we concluded that the proposed antenna can increase the performance level of SigFox wireless communications.

The antenna was tested in a real operating environment and its performance was compared with two reference antennas: one omnidirectional and one microstrip antenna, both designed for the 868 MHz frequency band.

This paper thus proposes a new antenna design that is able to increase the performance level of IoT applications by means of an original design. The antenna was designed, simulated, tested and evaluated in a real operating scenario and from the obtained results, it ensures a high level of performance that can be used in IoT applications specific to the 868 MHz frequency band regardless of the wireless communication protocol used.

## Data Availability

Not applicable.
